# Monomeric Flavanols Are More Efficient Substrates for Gut Microbiota Conversion to Hydroxyphenyl‐γ‐Valerolactone Metabolites Than Oligomeric Procyanidins: A Randomized, Placebo‐Controlled Human Intervention Trial

**DOI:** 10.1002/mnfr.201901135

**Published:** 2020-04-20

**Authors:** Wendy J Hollands, Mark Philo, Natalia Perez‐Moral, Paul W Needs, George M Savva, Paul A Kroon

**Affiliations:** ^1^ Food Innovation and Health Programme Quadram Institute Bioscience Norwich NR4 7UQ UK; ^2^ Core Research Services Quadram Institute Bioscience Norwich NR4 7UQ UK

**Keywords:** catechin, epicatechin, flavonoids, microbial metabolism, proanthocyanidins

## Abstract

**Scope:**

The majority of ingested flavanols reach the colon where they are catabolized by the microbiota to form hydroxyphenyl‐γ‐valerolactones (HGVLs). It is not known if the HGVLs are catabolic products of monomeric (epi)catechins (EPC), oligomeric procyanidins (OPCs), or both. Using data from a randomized, double‐blind, placebo‐controlled crossover trial the relative contributions of catechins and OPC to the bioavailable pool of HGVLs are estimated.

**Methods and results:**

Participants ingested an apple extract once daily for 28 days that delivered the following: i) 70 mg EPC and 65 mg OPC (low dose EPC), ii) 140 mg EPC and 130 mg OPC (high dose EPC), iii) 6 mg EPC and 130 mg OPC (OPC), and iv) a placebo control. Urine is collected over a 24‐h period before and after treatments. The median urinary excretion of HGVLs after ingestion of the high dose EPC is tenfold higher than that excreted after ingestion of the OPC that provided an equivalent dose of PC. Approximately 22% of catechins are converted to HGVLs in contrast to PC, for which there is limited conversion.

**Conclusion:**

Monomeric catechins are efficiently converted to derived HGVLs that are absorbed and excreted in human urine, whereas oligomeric PCs are much less efficiently converted.

## Graphical abstract

Apples are a rich dietary source of the flavanols (‐)‐epicatechin and procyanidins. There is limited information regarding the type of flavanol that forms hydroxyphenyl‐γ‐valerolactones (HGVLs). After 28 days ingestion of an apple extract containing different doses of epicatechin and procyanidins, it is shown that ≈22% of catechins are converted to HGLVs compared with procyanidins, for which there is limited conversion.

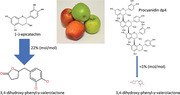

## 1 Introduction

Human intervention trials with flavanol‐rich foods and beverages have shown improvements in biomarkers of cardiovascular disease (CVD) after ingestion.^[^ ^1^, ^2^, ^3^ ^]^ Cocoa and some apple varieties are rich dietary sources of both monomeric flavanols, ((‒)‐epicatechin (EC) and (+)‐catechin) and procyanidins (PC); oligomers/polymers of epicatechin and catechin. Yet whether the observed beneficial effects of consuming flavanol‐rich foods are due to the monomeric flavanols or the oligomeric/polymeric PC is not known.

To exert the physiological effects observed in human intervention trials a compound must be bioavailable. Like all flavonoids, absorption and metabolism of flavanols is influenced by both chemical structure and molecular weight. The complex absorption and metabolism of low molecular weight monomeric catechins has been well described.^[^ ^4^ ^]^ After ingestion, monomeric catechins are predominantly absorbed in the small intestine after which they are metabolised by phase II conjugating enzymes to appear in plasma as sulfated and glucuronidated derivatives of epicatechin and methylepicatechins. In humans, circulating metabolites of monomeric (epi)catechins have been reported to reach peak plasma concentrations of 3.5–9 µmols L^−1^ within 30–90 min of ingestion with corresponding urinary excretion rates ranging between 88 and 200 µmols per day, depending upon the dose of (epi)catechins ingested.^[^ ^5^ ^]^ Monomeric (epi)catechins that have not been subject to absorption in the small intestine, or otherwise effluxed back into the small intestine from the liver through bile excretion, will reach the colon.

The metabolic fate of higher molecular weight oligomeric/polymeric PC on the other hand is less well understood. While PC have been shown to be stable during gut transit in humans,^[^ ^6^ ^]^ they are poorly absorbed with only PC dimers detected in human plasma and at concentrations ≈100‐fold lower when compared with the monomeric (epi)catechins.^[^ ^7^, ^8^, ^9^ ^]^ For example, after ingestion of a cocoa drink providing 2.1 mg kg^−1^ BW of monomeric catechins and 9.91 mg kg^−1^ BW of PCs (ranging from dp2–10), by 12 individuals in a randomized cross‐over trial, only 4 nmol L^−1^ of PC B2 was detected in plasma, compared with 823 nmol L^−1^ of EC metabolites, 2 h after ingestion.^[^ ^8^ ^]^ Likewise, after ingestion of a capsule containing 1 mg kg^−1^ BW of EC and 1 mg kg^−1^ BW of PC B1 by six individuals in a randomized cross‐over trial, only 1.9 ng mL^−1^ of PC B1 was detected in plasma, compared with ≈170 ng mL^−1^ of EC metabolites.^[^ ^9^ ^]^ These findings are supported by a human intervention trial with otherwise healthy ileostomy subjects. After ingestion of apple juice containing 157 mg L^−1^ of PC, more than 90% of the ingested PC was recovered in ileostomy effluent, indicating that PC are likely to reach the colon intact under normal physiological conditions.^[^ ^10^ ^]^

Oligomeric/polymeric PC and monomeric (epi)catechins that are not absorbed in the small intestine reach the colon where they are catabolized by the gut microbiota to form phenolic acids and hydroxyphenyl‐γ‐valerolactones (HGVLs). After flavanol ingestion, significant amounts of glucuronide and sulfate conjugates of 5‐(3′,4′‐dihydroxyphenyl)‐γ‐valerolactone and 5‐(3′‐methoxy‐4′‐hydroxyphenyl)‐γ‐valerolactone have been detected in plasma and urine.^[^ ^11^, ^12^ ^]^ However, how each flavanol contributes to the formation of these HGVLs is largely unknown. Recently, Ottaviani et al. investigated the formation of HGVLs in humans from various monomeric and oligomeric flavanols.^[^ ^13^ ^]^ They reported that the oral intake of (‒)‐epicatechin or (+/‒)‐catechin (dose = 120 µmol) and an equimolar amount of procyanidin B2, resulted in similar amounts of urinary excretion (≈60% of intake on a molar basis) of HGVL 3′/4′‐sulfates and 3′/4′‐glucuronides. The authors did not assess the relative contribution of PC with a higher degree of polymerisation (dp > 2).

Understanding the bioavailability of monomeric and oligomeric flavanols is important for interpreting the cardio‐protective effects of flavanol rich foods. Procyanidin‐rich foods such as cocoa products and apples contain significant quantities of oligomeric PC (dp3‐10+) but relatively small quantities of PC dimers (dp2). Our aim was to estimate the relative contributions of monomeric (epi)catechins versus oligomeric PC (dp2‐10) to the bioavailable pool of HGVLs. We used data from a cross‐over study into the effects of monomeric (epi)catechins and PC on systolic blood pressure and other cardiometabolic risk markers for CVD.^[^ ^14^ ^]^

## 2 Experimental Section

### 2.1 Chemicals and Reagents

Formic acid, dimethylformamide (DMF), acetonitrile, taxifolin, and ammonium acetate were purchased from Sigma‐Aldrich (Poole, UK). The (‒)‐epicatechin reference standard was purchased from Toronto Research Chemicals (Canada). The reference standards 5‐(3′,4′‐dihydroxyphenyl)‐γ‐valerolactone (3,4DHVL) and 5‐(3′‐methoxy‐4′‐hydroxyphenyl)‐γ‐valerolactone (4H3MVL) were synthesized in‐house as described by Chang et al.^[^ ^15^ ^]^ The phase‐2 conjugates, specifically 5‐(3′‐hydroxyphenyl)‐γ‐valerolactone‐4’‐glucuronide (3HGV4‐glucuronide), 5‐(4′‐hydroxyphenyl)‐γ‐valerolactone‐3’‐glucuronide (4HVL3‐glucuronide), 5‐(3′‐hydroxyphenyl)‐γ‐valerolactone‐4’‐sulfate (3HGV4‐sulfate), and 5‐(4′‐hydroxyphenyl)‐γ‐valerolactone‐3’‐sulfate (3HGV4‐sulfate) were synthesized in‐house as described in the Supporting Information.

### 2.2 Subjects and Study Design

The study was a randomized, double‐blind, placebo controlled, four‐treatment crossover trial and is described in detail elsewhere.^[^ ^14^ ^]^ Briefly, the aim of the parent trial was to investigate the acute and chronic effects of ingesting two different doses of monomeric (epi)catechins (70 and 140 mg) and type of isolated apple derived flavanols (catechins vs PC) on systolic blood pressure and other cardiometabolic risk markers for CVD. Urine was collected from participants before and after ingestion of the treatments to assess the bioavailability of monomeric (epi)catechins and oligomeric PC. The trial was conducted at the Quadram Institute Bioscience, Norwich, UK between August 2014 and March 2016. Forty‐two participants (15 men and 27 women) aged 50 years and older with moderately elevated systolic blood pressure completed the trial. All participants gave written informed consent before taking part in the trial and the study protocol was approved by the Norfolk Research Ethics Committee (Ref: 13/EE/0393). The trial is registered at http://clinicaltrials.gov (Identifier: NCT02013856).

### 2.3 Dosage Information

Participants ingested the following daily for 28 days: i) an apple extract delivering a daily dose of 70 mg monomeric flavanols and 65 mg PC (from this point forward referred to as low dose EPC), ii) an apple extract delivering a daily dose of 140 mg monomeric flavanols and 130 mg PC (from this point forward referred to as high dose EPC), iii) an apple extract significantly depleted of monomeric flavanols but delivering a daily dose of 130 mg PC (from this point forward referred to as OPC), and iv) a placebo control. Extracts were encapsulated, and two capsules (together providing the daily dose) were ingested once daily for 4‐weeks. For 24‐h prior to and for the duration of the 28‐day treatment period, participants were asked to exclude from their diet some foods that were particularly rich in flavanols (e.g., dark chocolate and cocoa) and to limit others to a level that would support compliance (e.g., tea). A limited number of food sources were completely excluded from the diet for the 24‐h urine collection period. To aid compliance, participants were given a list of prohibited and limited foods.

### 2.4 Assessment of Outcomes

Urine was collected over a 24‐h period before starting the treatment (baseline) and then again after 28 days of treatment. Completed 24‐h urine collections were weighed and total volume recorded. Sub‐samples of urine (5 mL) were then aliquoted into appropriate tubes and acidified with 2 m hydrochloric acid to reduce the pH to ≈4.5. Samples were then stored at −80 °C until batch analysis. Each treatment period was separated by a 1‐month wash‐out phase.

### 2.5 Preparation and Analysis of Apple Extracts

Analysis of the apple extracts has been described in detail elsewhere.^[^ ^14^ ^]^ In brief, the apple extracts were prepared from freeze dried apples that were further processed to produce i) an epicatechin rich extract containing around 30% w/w of monomeric catechins (90% (‒)‐EC, 10% (+)‐catechin) that retained some of the oligomeric PC and ii) an oligomeric PC‐rich extract that was significantly depleted of monomeric catechins. To determine the flavanol content of the apple extracts, samples were analyzed by normal phase HPLC (Agilent 1100) with separation of the analytes being achieved using a Luna Hilic column (150 × 2.0 mm; 3 µm) coupled with fluorescence detection as described previously.^[^ ^16^ ^]^ Flavanol composition of the treatments is shown in **Table** **1**.

**Table 1:** Composition (mg) of monomeric catechins and oligomeric procyanidins in high and low dose EPC and OPC treatments

Compound	High dose EPC	Low dose EPC	OPC
(–)‐Epicatechin	126 (434)	63 (217)	6 (22)
(+)‐Catechin	14 (48)	7 (24)	0
Sum of monomers	140 (482)	70 (241)	6 (22)
dp 2	80 (277)	40 (138)	10 (36)
dp 3	32 (109)	16 (55)	14 (49)
dp 4	11 (39)	6 (20)	22 (76)
dp 5	4 (15)	2 (7)	24 (82)
dp 6	2 (8)	1 (4)	19 (66)
dp 7	1 (4)	0.6 (2)	13 (44)
dp 8	0	0	10 (36)
dp 9	0	0	10 (36
dp 10	0	0	7 (26)
Sum of procyanidins	130 (452)	65 (226)	130 (450)

Values in parentheses are µmols equiv. dp = degree of polymerization. High and low dose EPC = 140/70 mg monomeric catechins + 130/65 mg PC, respectively; OPC = 6.5 mg monomeric catechins + 130 mg PC.

### 2.6 Identification and Quantification of (epi)Catechin and Hydroxyphenyl‐γ‐Valerolactone Metabolites in Urine

Urine samples (200 µL) were mixed with formic acid (10 µL), dimethylformamide (10 µL), and taxifolin (10 µL; 10 µg mL^−1^) and allowed to stand for 20 min at room temperature. Samples were then centrifuged (15 min; 13 000 RPM) and filtered (0.45 µm) prior to analysis. Samples were injected onto a Waters HSS T3 C18 column (100 mm × 2.1 mm; 1.7 µm) connected to an Agilent 1290 UPLC system coupled to a 6490‐triple quadrupole mass spectrometer (Agilent, UK). Samples were eluted at a flow rate of 0.4 mL min^−1^ and elution was achieved using an increasing gradient of solvent B (acetonitrile) from solvent A (50 mm ammonium acetate; pH 5.0) as follows: 0 min, 0%; 0.5, min 0%; 20 min, 18%; 30 min, 90%; 30.1 min, 0%; 35 min, 0%. The injection volume was 2 µL. The eluent passed through the mass spectrometer (MS) operated in the negative electrospray ionisation mode. The optimized MS operating parameters for each of the metabolites are described in **Table** **2**.

**Table 2:** Parameters for identification of (epi)catechin and HGVL metabolites in urine by LC‐MS/MS

Analyte	Precursor ion [*m*/*z*]	Product ion [*m*/*z*]	Dwell time	CE [v]	RT [min]
(‒)‐Epicatechin	289	245	20	11	21.7
(+)‐Catechin	289	245	20	11	19.0
(Epi)catechin‐di‐glucuronide	641	465	10	22	n.d.
Methyl (epi)catechin	303	259	10	30	21.3
Methyl (epi)catechin‐sulfate	382	303	10	30	n.d.
(Epi)catechin‐sulfate	369	289	10	25	20.8
(Epi)catechin‐methyl‐sulfate	383	303	10	30	21.5
(Epi)catechin glucuronide	465	289	10	30	16.1
(Epi)catechin‐sulfate‐glucuronide	545	465	10	22	16.5
(Epi)catechin‐di‐sulfate	413	253	10	30	18.1
3,4DHVL 3′‐sulfate	287	207	20	45	19.0
3,4DHVL 4′‐glucuronide	383	163	20	45	14.5
3,4DHVL 3′‐glucuronide	383	163	20	45	15.5
3,4DHVL	207	163	20	25	19.2
4H3MVL	221	162	20	25	22.8

3,4DHVL = 5‐(3′,4′‐dihydroxyphenyl)‐γ‐valerolactone; 4H3MVL = 5‐(4‐hydroxy‐3‐methoxyphenyl)‐γ‐valerolactone.

CE, collision energy; RT, retention time; n.d., not detected.

Ten chromatographic peaks were identified in the 24‐h urine samples collected before and after 28 days of treatment. Two of the peaks produced a *m*/*z* ratio of 207/163 and 221/162 which corresponded with the retention time of the authentic 3,4DHVL and 4H3MVL reference standards, respectively. The remaining eight post‐treatment chromatographic peaks generated the expected mass transitions for glucuronides and sulfates of (epi)catechin and HGVLs (see Table 2).

(Epi)catechin aglycones and conjugates and HGVL aglycones were quantified against matrix matched calibration standard curves of epicatechin and 3,4DHVL/4H3MVL, respectively, over the range 10 to 2250 ng mL^−1^ that were processed in the same manner as the participant urine samples. The response factors used to quantify urinary metabolites of (epi)catechin and HGVLs were derived from plots of the epicatechin, 3,4DHVL, and 4H3MVL/internal standard peak area ratio against the corresponding spiked standard concentration. HGVL conjugates were quantified using relative response factors (RRF) for 4HVL‐3’‐glucuronide, 3HVL‐4’‐glucuronide, 4HVL‐3’‐sulfate, and 3HVL‐4’‐sulfate that were synthesised in‐house. Matrix‐matched calibration curves were prepared for 3,4DHVL and these four conjugates were analyzed in the same way as samples. The RRF values were as follows: 4HVL‐3′‐glucuronide—2.946; 3HVL‐4′‐glucuronide—3.607; 4HVL‐3′‐sulfate—0.246; 3HVL‐4′‐sulfate—0.246. Estimates of urinary excretion of the metabolites was obtained by multiplying the estimated urine concentrations with the total volume of urine excreted in the 24‐h collection period. The reference standard curves were linear with a correlation coefficient > 0.98. The limit of detection (LOD) and limit of quantification (LOQ) were 5 and 10 ng mL^−1^, respectively.

### 2.7 Statistical Analysis

Distributions of (epi)catechins and HGVLs in 24‐h collections were highly skewed, and so are described using medians and quartiles.

The distribution of specific (epi)catechins and HGLVs (as a proportion of the epicatechin and HGLV group totals) were calculated for each individual and then averaged over the sample.

To estimate the relationship between administered doses of monomeric flavanols and oligomeric PC and subsequent excretion of HGVL metabolites, a nonlinear regression model was estimated using the Bayesian Regression Models using Stan (brms) package in R statistical software.^[^ ^17^, ^18^, ^19^ ^]^ This model was developed theoretically and through descriptive analysis of the data, and incorporated the expected linear relationship between dose and total metabolite excretion, with a multiplicative error term incorporating a group effect corresponding to participant to account for within‐participant correlation. Two different residual variances estimated for i) the “high dose” and “low dose” post‐treatment excretions and ii) all other groups, reflecting slightly different variances observed when plotting metabolite excretion on a logarithmic scale. The model and all parameter estimates are fully described in Supporting Information.

## 3 Results

### 3.1 Study Participants

The baseline demographics of the 42 study participants were (mean ± SD): Age, 63 ± 7 year; body weight, 73 ± 13 kg; BMI, 25.9 ± 3.1 kg m^−2^. No serious adverse events were reported during the trial. Compliance to treatments was assessed by use of a capsule checklist and counting the number of unused capsules returned at the end of each treatment period. Overall compliance to treatments was high with >99% of capsules ingested across all four treatments when assessed as a proportion of the intended total.

It was apparent from analysis of outcome data that two participants did not consume the high‐dose EPC capsules on day 28 of this treatment, as evidenced by almost undetectable levels of (epi)catechin metabolites in the 24‐h urine collection and correspondingly low HGVLs (lowest ranked of all 42 participants). These two participants were not low urinary excretors of (epi)catechin metabolites on the low dose EPC treatment. On this basis, the data arising from the high dose EPC treatment for these two participants were excluded from the current analysis.

On three occasions urine was not collected, this data is considered to be missing at random, and each analysis uses all available data.

### 3.2 Urinary Excretion of Epicatechin and Hydroxyphenyl‐γ‐Valerolactones Metabolites

**Figure 1:**
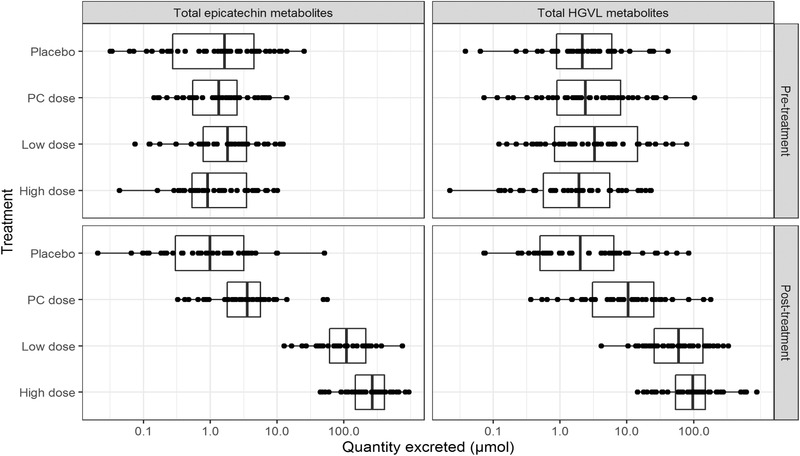
Variability in pre‐ and post‐treatment urinary excretion of (epi)catechin and HGVL metabolites. High‐ and low‐dose EPC = 140/70 mg monomeric catechins + 130/65 mg PC, respectively; OPC = 6.5 mg monomeric catechins + 130 mg PC.

Some urinary excretion of (epi)catechin and HGVL metabolites was evident in the pre‐treatment 24‐h urine collections for all participants, and as expected this did not differ between treatment groups. Median urinary excretion of total (epi)catechin metabolites in pre‐treatment samples was 1.4 µmol (IQR = 0.5 to 3.6), while median excretion of HGVL metabolites was 2.1 µmol (IQR = 0.8 to 6.8). As suggested by wide inter‐quantile range, pre‐treatment excretion of (epi)catechin metabolites and HGVL metabolites were extremely variable across participants (**Figure** **1** and **Table** **3**).

**Table 3:** Post‐treatment differences in urinary excretion of (epi)catechin and HGVL metabolites 28 days after daily ingestion of the high‐ and low‐dose EPC and OPC treatments and placebo

	High dose EPC	Low dose EPC	OPC	Placebo
(+) Catechin	0.0	0.0	0.0	0.0
(‒)‐Epicatechin	0.0	0.0	0.0	0.0
(Epi)catechin‐di‐glucuronide	0.0	0.0	0.0	0.0
Methyl (epi)catechin	0.0	0.0	0.0	0.0
Methyl (epi)catechin‐sulfate	0.0	0.0	0.0	0.0
(Epi)catechin‐sulfate	110.5 (44.8–170.8)	53.7 (15.3–95.2)	2.0 (0.8–3.1)	0.3 (0.1–1.0)
(Epi)catechin‐methyl‐sulfate	74.6 (44.9–129.8)	36.9 (24.7–63.0)	1.4 (0.6–2.1)	0.5 (0.1–1.2)
(Epi)catechin glucuronide	45.8 (29.3–77.8)	19.2 (10.2–35.1)	0.1 (0.1–0.4)	0.1 (0.0–0.2)
(Epi)catechin‐sulfate‐glucuronide	13.8 (7.2–23.6)	4.0 (3.0–9.4)	0.1 (0.0–0.2)	0.0 (0.0–0.1)
(Epi)catechin‐di‐sulfate	0.57 (0.2–1.5)	0.7 (0.2–1.5)	0.0	0.0
Median of (epi)catechin metabolites	267.2 (147.8–405.9)	109.3 (61.3–213.3)	3.6 (1.8–5.7)	1.0 (0.3–3.2)
3,4DHVL 3′‐ sulfate	38.4 (20.1–74.8)	25.1 (13.9–62.5)	5.2 (1.7–16.3)	1.3 (0.3–4.4)
3,4DHVL 4′‐glucuronide	16.0. (7.9–32.1)	7.6 (3.1–20.8)	1.2 (0.4–3.6)	0.3 (0.1–1.0)
3,4DHVL 3′‐glucuronide	36.0 (14.9–56.3)	14.4 (6.4–35.1)	2.0 (0.8–3.4)	0.3 (0.1–1.5)
3,4DHVL	0.6 (0.3–1.1)	0.4 (0.2–0.6)	0.1 (0.0–0.2)	0.0 (0.0–0.1)
4H3MVL	0.0	0.0	0.0	0.0
Median of HGVL metabolites	99.9 (53.9–153.3)	59.1 (26.3–146.9)	10.8(3.1–26.9)	2.2 (0.5–6.7)

Data are expressed as median (upper quartile and lower quartile) in µmols 24 h^−1^ (*n* = 40 participants for high dose EPC and 42 participants for low dose EPC and OPC treatments and placebo). High and low dose EPC = 140/70 mg monomeric catechins + 130/65 mg PC, respectively; OPC = 6.5 mg monomeric catechins + 130 mg PC. 3,4DHVL = 5‐(3’,4’‐dihydroxyphenyl)‐γ‐valerolactone; ‐4H3MVL = 5‐(4‐hydroxy‐3‐methoxyphenyl)‐γ‐valerolactone

### 3.3 Urinary Excretion of EC Metabolites

(Epi)catechin was excreted in urine almost exclusively as glucuronides and sulfates of methylated and non‐methylated (epi)catechins. The median absolute quantity of (epi)catechins excreted over the 24‐h period corresponding with the last day (28‐day) of consumption was 267.2 (IQR = 147.8 to 405.9) and 109.3 (IQR = 61.3 to 213.3); high and low dose EPC treatments, respectively. Following the OPC treatment participants excreted 3.6 µmol (IQR = 1.8 to 5.7) (epi)catechin phase‐2 metabolites (Table 3).

Urinary excretion of the individual (epi)catechin metabolites as a proportion of the total epicatechins were very similar between the low and high dose EPC treatments. (Epi)catechin sulfates and (epi)catechin methyl sulfates were the most abundant metabolites in 24‐h urine samples accounting for ≈39% and 36% of the total, respectively. (Epi)catechin glucuronides and (epi)catechin sulfoglucuronides accounted for around 19% and 6%, respectively. Very small amounts of (epi)catechin disulfate were detected accounting for less than 1% of the total excreted.

### 3.4 Urinary Excretion of HGVL Metabolites

The major HGVL metabolites in urine were the 4′‐glucuronide, 3′‐glucuronide, and 3′‐sulfate of 3,4DHVL and 3′‐sulfate of 3,4,5THVL. Median urinary excretion of HGVL metabolites over the entire 24‐h period after 28 days of consumption was 99.9 (IQR = 53.9 to 153.3) and 59.1 (IQR = 26.3 to 146.9) µmol; high and low dose EPC treatments, respectively. Median urinary excretion of HGVL metabolites after ingestion of the OPC treatment was 10.8 (IQR = 3.1 to 26.9) µmol; tenfold lower compared to the high dose EPC treatment that provided an equivalent dose of PC (Table 3).

Urinary excretion of the individual HGVL metabolites as a proportion of the total HGVLs were also very similar between the high and low dose EPC treatments. When combined, the 3,4DHVL 3′ and 4′‐glucuronides accounted for around 55% of the total HGVLs. This was followed by 3,4DHVL 3′‐sulfate (44%), 3,4DHVL (1%), and 4H3MVL (0.5%).

### 3.5 Source of Hydroxyphenyl‐γ‐Valerolactones

Each treatment provided both monomeric catechins and oligomeric PC in known amounts (Table 1). By modeling the relationship between administered flavanol doses in micromoles and total HGVL production, we estimated the conversion factors for production of HGVLs from monomeric catechins and from oligomeric PC. Estimating this model suggests that for each 1 µmol increase in monomeric catechin leads to a 0.22 µmol increase in HGVLs (Bayesian 95% credible interval = 0.15 to 0.31). However, there was no significant effect of PCs on HGVL production; a 1 µmol increase in PC was associated with only a 0.005 µmol increase in HGVLs (Bayesian 95% credible interval = −0.002 to 0.013). The full model is described in Supporting Information.

### 3.6 Inter‐Individual Variation

The inter‐individual variation in urinary excretion of total (epi)catechin and HGVL metabolites after 28 days of ingestion of all three treatments was very high (descriptive statistics shown in **Table** **4**). The highest and lowest HGLV excretions observed in our sample of 40 participants who took the high dose were 14.7 versus 881.8 mmol, a 60‐fold difference. This variation is mainly attributable to variation between participants as opposed to error in measurement or day‐to‐day variation within participants, as indicated by the high correlation between HGLV excretion after high dose and low dose treatments for each participant (Spearman's rank correlation = 0.70). There was a similarly high correlation for high dose and low dose (epi)catechin excretion (Spearman's rank correlation = 0.68).

**Table 4:** Inter‐individual variation in 24‐h urinary excretion of total (epi) catechin and HGVL metabolites 28 days after daily ingestion of the high‐ and low‐dose EPC and OPC treatments and placebo

Variation parameters	High dose EPC	Low dose EPC	OPC	Placebo
(Epi)catechins (µmols 24 h^−1^)				
Highest value	939.7	749.9	56.6	51.0
Lowest value	44.5	12.7	0.3	0.0
Mean	303.7	149.1	6.3	3.0
Median	267.2	109.3	3.6	1.0
SD	216.4	130.2	11.0	7.9
SEM	34.2	20.1	1.7	1.2
% CV	71.2	87.3	175.9	262.4
HGVLs (µmols 24 h^−1^)				
Highest value	881.8	335.3	182.9	85.9
Lowest value	14.7	4.3	0.38	0.0
Mean	155.1	88.7	22.1	8.7
Median	99.9	59.1	10.8	2.2
SD	184.3	84.3	36.8	17.2
SEM	29.1	13.0	5.7	2.6
% CV	118.8	95.0	166.4	197.3

Data are expressed as µmols 24 h^−1^ (*n* = 40 participants for high dose EPC and 42 participants for low dose EPC and OPC treatments and placebo). High and low dose EPC = 140/70 mg monomeric catechins + 130/65 mg PC, respectively; OPC = 6.5 mg monomeric catechins + 130 mg PC.

## 4 Discussion

The aim of this analysis was to estimate the relative contributions of monomeric catechins and oligomeric PC (dp2‐10) to the bioavailable pool of HGVLs. In this randomized, four‐arm cross‐over trial we have shown that PC are not a significant source of gut microbiota‐derived HGVLs that are absorbed and excreted in human urine, and that the predominant source of HGVLs are the monomeric flavanols such as epicatechin. Furthermore, our trial contributes to the growing body of evidence that individual differences in composition of the gut microbiota may differentially affect the formation of microbial‐derived metabolites, as evidenced by the high individual variation in urinary excretion of HGVLs across three flavanol doses, and the correlation of excretion within individuals such that those with higher excretion of HGVLs at one dose typically had higher levels at other doses on other occasions. The total 24‐h urinary yield of HGVLs reported here was 99 µmols from the 482 µmols of monomeric catechins in the high dose EPC group (20% yield) which is comparable to the report of Anesi et al.^[^ ^20^ ^]^ in which the authors fed 400 g apple containing 775 µmols of monomeric catechins to 11 individuals and reported a 24‐h urinary yield of 197 µmols (25% yield).

The absorption and metabolism of monomeric catechins has been extensively investigated. In humans, monomeric catechins are rapidly absorbed in the small intestine. After absorption they undergo extensive metabolism in both the small intestine and liver to form sulfated, glucuronidated, and methylated derivatives of (epi)catechin. Monomeric catechins have been reported to reach peak plasma concentrations within 1–2 h of ingestion,^[^ ^5^, ^11^, ^14^ ^]^ with urinary excretion rates reported to be around 40–60% of dose.^[^ ^5^ ^]^ Here we found median urinary excretion rates of (epi)catechin structurally‐related metabolites to be 51% of dose (Bayesian 95% credible interval 39–66%, see Supporting Information for details of statistical model) which is in keeping with this literature estimate.

The metabolic fate of oligomeric PC is still under consideration. To date, results from human and animal intervention studies have shown intact oligomeric PC dimers (and to a lesser extent trimers) to be absorbable.^[^ ^7^, ^8^, ^9^, ^21^ ^]^ After giving apple procyanidins to rats (dose = 1 g kg^−1^), PC dimer B2 and trimer C1 were detectable in plasma at concentrations of 0.4 and 0.14 µm, respectively.^[^ ^21^ ^]^ Similarly, PC B2 was detected in human plasma at a concentration of 0.041 µm after ingestion of cocoa (dose = 0.375 g kg^−1^ BW).^[^ ^7^ ^]^ While PC dimers are shown to be bioavailable, the amount absorbed does appear to be somewhat limited. For example, in an in situ perfusion study of male Wister rats fed purified PC dimers and monomeric epicatechin, absorption of the PC dimers was shown to be only 5–10% of that of monomeric epicatechin. Moreover, the dimers were neither conjugated or methylated indicating that phase 2 metabolism of absorbable PC does not occur, unlike that of epicatechin which was partly methylated and completely conjugated.^[^ ^22^ ^]^ This may be explained in part at least by differences in the processes of absorption and phase‐2 conjugation. There is evidence that PC are preferentially absorbed across the intestinal epithelium via the paracellular pathway,^[^ ^23^ ^]^ whereas it has been shown that (–)‐epicatechin is phase‐2 conjugated and is therefore absorbed via the trans‐epithelial route.^[^ ^24^ ^]^ To our knowledge PC with a degree of polymerization > 3 have not been shown to be absorbable in humans.

It is estimated that ≈70% of flavanols enter the colon unabsorbed.^[^ ^11^, ^25^ ^]^ Here, the colonic microbiota is essential for facilitating bioavailability by converting flavanols into phenolic compounds, such as HGVLs, that are more efficiently absorbed by intestinal epithelial cells. There is a paucity of information regarding the type of flavanol that forms HGVLs. Previously, Ottaviani and colleagues reported that epicatechin and PC ingestion did not yield significantly different amounts of HGVLs in urine (43% and 54%, respectively) after human volunteers ingested a mimetic cocoa powder drink containing epicatechin alone or a PC‐rich low epicatechin drink (dp2‐10). However, the authors report that the amount of PC ingested was five times higher than that of epicatechin, concluding that epicatechin may still be a more significant source of HGVLs than PC.^[^ ^8^ ^]^ In our trial, the median urinary excretion of HGVL metabolites after ingestion of the high dose EPC treatment was tenfold higher than that excreted after ingestion of the OPC treatment that provided an equivalent dose of oligomeric PC (99 vs 11 µmol per day; high dose EPC, OPC treatments, respectively; dose = 452 µmols PC). These data strongly indicate that monomeric catechins are indeed significantly more efficient substrates for gut microbiota conversion to HGVLs than oligomeric PC. Moreover, since all three treatments in our trial provided both monomeric (epi)catechins and oligomeric PC (Table 1), it was possible to estimate the conversion factors for the production of HGVLs from monomeric (epi)catechins and from oligomeric PC. In doing so, we estimate that on average 22% of monomeric (epi)catechins are converted to HGVLs (although this varied enormously between individuals) in contrast to PC oligomers for which there was very limited conversion. Since the dose of PC fed to our participants was substantially smaller than reported for cocoa procyanidins for which there was evidence of a significant contribution to HGVL production,^[^ ^8^ ^]^ it appears that procyanidins can be converted to HGVLs but much less efficiently than monomeric epicatechin. However, it is possible that the lowest molecular weight PC (e.g., dimers‐like PC B1) are more efficiently converted into HGVLs than higher dp PC. Indeed, there is evidence from studies using in vitro colonic fermentation and from a human feeding study that suggest the efficacy of conversion of PC dimers such as B1 to HGVLs is much higher than for higher dp oligomers.^[^ ^9^, ^26^ ^]^

So, what then is the metabolic fate of the majority of PCs? It was not within the remit of our trial to identify and quantify other catabolites of PCs, but several degradation pathways have been proposed. First, 2‐(3′,4′‐dihydroxyphenyl) acetic acid could result from direct degradation of the upper unit of procyanidin B2 as illustrated by Mena et al in their review of the formation, bioavailability and pharmacokinetics of valerolactones and valeric acids.^[^ ^27^ ^]^ Second, early in vitro studies have also identified 5‐(2′,4′‐dihydroxy) phenyl‐2‐ene valeric acid and 5‐(3′,4′‐dihydroxyphenol) valeric acid after incubation of procyanidin B2 with human fecal bacteria but not after incubation with (‒)‐epicatechin.^[^ ^28^ ^]^ These data indicate that they are likely to be unique catabolites of procyanidin dimers.

It is widely accepted that factors such as age, gender, and dietary habits may influence the production of metabolites after ingestion of flavanol rich foods and beverages and that inter‐individual differences in absorption and metabolism of flavanols may have an impact on the potential health benefits. Since it has been estimated that ≥70% of ingested flavanols reach the colon unabsorbed,^[^ ^11^, ^25^ ^]^ the colonic microbiota is then an important consideration when assessing inter‐individual variation in flavanol metabolism. In our study, we observed substantial inter‐individual differences in urinary excretion of HGVLs, by high correlations within individuals, suggesting that this variation is caused mainly by participant specific factors that were stable over the course of the study as opposed to measurement error or day‐to‐day variation. This is in keeping with several other human studies assessing the bioavailability of HGVLs after ingestion of flavanol‐rich foods and beverages.^[^ ^29^, ^30^ ^]^ For example, after ingestion of green tea by 16 participants in a single blinded cross‐over trial, the authors report a co‐efficient of variation in urinary excretion of HGVL metabolites between 200% and 300%.^[^ ^29^ ^]^ These data, like that of the trial reported here, indicate that individual differences in composition of the gut microbiota may differentially affect the formation of microbial‐derived metabolites.

A strength of our study was the well‐controlled, cross‐over approach. This has the advantage in that participants act as their own control, thus minimizing biological and methodological variation. Since the OPC treatment was almost devoid of (epi)catechins and delivered an equivalent dose of PC as the high dose EPC treatment, we were able to readily distinguish between the respective contribution of monomeric catechins and oligomeric PC to the bioavailable pool of HGVLs. Study limitations include the fact that other microbial catabolites of the ingested flavanols were not identified and quantified which may have provided some meaningful insight into the degradation of PCs.

In conclusion, our data show that monomeric catechins are efficiently converted to gut microbiota‐derived metabolites such as HGVLs that are absorbed and excreted in human urine, whereas oligomeric PCs are much less efficiently converted.

## Conflict of Interest

The authors declare no conflict of interest.

## Author Contributions

P.A.K. and W.J.H. designed the study; W.J.H. and N.P.‐M. were responsible for participant recruitment and day‐to‐day management of the trial; P.W.N. synthesized the reference standards used for quantification of HGVLs; M.P. undertook MS‐based quantification of (epi)catechin and HGVL metabolites; G.M.S. statistically analyzed data; W.J.H., P.A.K., and G.M.S. wrote the manuscript with contributions from the other authors.

## Supporting information

Supporting Information

Supporting Information
