# Supplementary material for: Monomeric Flavanols Are More Efficient Substrates for Gut Microbiota Conversion to Hydroxyphenyl‐γ‐Valerolactone Metabolites Than Oligomeric Procyanidins: A Randomized, Placebo‐Controlled Human Intervention Trial
Source: Mol Nutr Food Res. 2020 Apr 20;64(10):1901135. doi: 10.1002/mnfr.201901135 (PMC7378946; doi:10.1002/mnfr.201901135)
Supplement: Supplementary file 1 — Supporting Information [file MNFR-64-1901135-s002.docx]

**Supplementary file**

**Detail of statistical model for HGVL and catechin excretion as a function of monomeric flavonoid and doses.**

Each treatment provides procyanidins and monomeric catechins in known amounts (see **table 1**). Each participant returned up to eight observations, corresponding to four pre-treatment measurement (no additional PC and MC beyond normal diet) and four observations taken after 28 days after each treatment.

**Statistical model specification**

Our statistical model for HGVL excretion assumes a linear relationship between ingested doses and excreted HGVLs, with a multiplicative error term to explain variation between and within participants. A multiplicative error is used because the distribution of HGVLs within each treatment group appears normally distributed following a logarithmic transformation.

Descriptive analysis also suggests that the errors within individuals are correlated, that is individuals with high HGVL excretion under one treatment are also high excreters under other conditions. Hence the need for a multilevel model including error terms at both the patient and observation level.

So, the model used to estimate the contribution of PC and MC to HGLV excretion was:

$$y_{ij}=\left( a+b_{1}\times PC_{i}+b_{2}\times MC_{i} \right)^{\eta_{j}+ \epsilon_{ij}}$$

Where $y_{ij}$ is the HGLV excretion for patient *j* under treatment *i*, $PC_{i}$ and $MC_{i}$ are the doses of PC and MF supplied by treatment $i$*,* $a$ is the geometric mean of HGLV with no additional PC or MF, $b_{1}$ and $b_{2}$ are the effect of each µMol PC and MC on µMol HGLV excretion respectively, $\eta_{j}$ is the multiplicative effect of participant *j* on the outcome, and $\epsilon_{ij}$ is the residual multiplicative error associated with observation *ij*. The variance components $\eta$ and $\epsilon$ are both drawn from normal distributions, with standard deviations $\sigma_{\eta}$ and $\sigma$ respectively. The residual standard deviation, $\sigma_{\epsilon}$, was allowed to vary depending on treatment, following the observation that the log-transformed data was more variable for the placebo and PC treatments compared to the LD and HD.

**Computation**

The model was estimated on the logarithmic scale, using the brms package version 2.10.0 in R version 3.6.1, with default prior distributions for all unknown parameters. Four chains were used, each with 5000 warmup iterations and 5000 main iterations.

**Results**

The posterior mean estimates of model parameters, with posterior standard deviation and credible interval are shown in **supplementary table 1**. This shows that the average excretion in the baseline condition (placebo plus pre-treatment observations) was $a$*=*2.5 µMol HGLV, plus $b_{2}$=0.22 µMol for each 1 µMol of monomeric catechins in the administered treatment. There was no substantial effect of PC on HGVL production ($b_{1}=$ 0.005 µMol HGLV per µMol PC).

All r-hat values were 1.00 indicating good convergence. The model fit to the data was determined by comparing geometric means of posterior random draws from the model to the observed geometric mean values (**table 3**). These fitted values were very close to the observed at the PC dose and placebo doses, were slightly under the observed values at the high dose but slightly lower at the low dose, suggesting that the response might be slightly attenuated at the highest doses, however there was no strong evidence for quadratic term when tested in sensitivity analysis.

Finally, the excretion of catechins was modelled using the same model (estimates shown in **supplementary table 2**, compared with observed value in **supplementary table 3**). This showed that 0.51 µMol catechins excreted per µMol HGLV, but no evidence for effect of PC consumption on catechin excretion.

**Sensitivity analyses**

Additional models were estimated using different parameterisations for the errors, in particular (i) assuming common error standard deviation for all treatments and (ii) adding an additional random effect into the linear component to allow for between participant variation in background diet. Neither of these made any difference to the estimates of $b_{1}$ or $b_{2}$. Two final models were estimated allowing a non-linear effect of MC on HGVL through adding but there was no strong evidence of a non-linear relationship for either outcome effect and so these was not included in the final model.

**Supplementary table 1**: Estimates from Bayesian non-linear model of µMol HGLV excretion.

|  | Posterior mean | Posterior standard deviation | Credible interval | |
| --- | --- | --- | --- | --- |
|  |  |  | Q2.5 | Q97.5 |
| a (Baseline) | 2.502 | 0.452 | 1.740 | 3.512 |
| b_2_ (MF ingested, per µMol) | **0.220** | **0.040** | **0.153** | **0.308** |
| b_1_ (PC ingested, per µMol) | 0.005 | 0.004 | -0.002 | 0.013 |
| log(σ_ε_) (HD treatment) | -0.355 | 0.148 | -0.643 | -0.062 |
| log(σ_ε_) (LD vs HD) | -0.139 | 0.199 | -0.533 | 0.248 |
| log(σ_ε_) (PC vs HD) | 0.402 | 0.201 | 0.010 | 0.801 |
| log(σ_ε_) (placebo vs HD) | 0.537 | 0.170 | 0.201 | 0.869 |
| σ_η_ | 1.01 | 0.13 | 0.78 | 1.29 |

**Supplementary table 2**: Estimates from Bayesian non-linear model of µMol (epi)catechin excretion.

|  | Posterior mean | Posterior standard deviation | Credible interval | |
| --- | --- | --- | --- | --- |
|  |  |  | Q2.5 | Q97.5 |
| a (Baseline) | 1.266 | 0.182 | 0.948 | 1.665 |
| b_2_ (MF ingested, per µMol) | **0.510** | **0.067** | **0.393** | **0.657** |
| b_1_ (PC ingested, per µMol) | -0.020 | 0.003 | -0.026 | -0.015 |
| log(σ_ε_) (HD treatment) | -0.712 | 0.172 | -1.065 | -0.388 |
| log(σ_ε_) (LD vs HD) | 0.182 | 0.233 | -0.256 | 0.654 |
| log(σ_ε_) (PC vs HD) | 0.514 | 0.218 | 0.096 | 0.956 |
| log(σ_ε_) (placebo vs HD) | 0.904 | 0.187 | 0.550 | 1.289 |
| σ_η_ | 0.74 | 0.10 | 0.57 | 0.96 |

**Supplementary table 3**: Modelled and observed geometric means of HGLVs and catechins under each treatment.

|  | HGLVs | | Catechins | |
| --- | --- | --- | --- | --- |
|  | Modelled | Observed | Modelled | Observed |
| High dose | 100.7 | 93.72 | 218.06 | 232.05 |
| Low dose | 51.9 | 54.31 | 114.28 | 104.02 |
| PC dose | 8.60 | 8.37 | 3.27 | 3.22 |
| Placebo / pre-treatment | 2.29 | 2.27 | 1.20 | 1.19 |
