# Supplementary material for: Monomeric Flavanols Are More Efficient Substrates for Gut Microbiota Conversion to Hydroxyphenyl‐γ‐Valerolactone Metabolites Than Oligomeric Procyanidins: A Randomized, Placebo‐Controlled Human Intervention Trial
Source: Mol Nutr Food Res. 2020 Apr 20;64(10):1901135. doi: 10.1002/mnfr.201901135 (PMC7378946; doi:10.1002/mnfr.201901135)
Supplement: Supplementary file 2 — Supporting Information [file MNFR-64-1901135-s001.docx]

We would like to thank Dr. I.J. Colquhoun for his help with interpretation of 2D nmr data.

**Synthesis of valerolactone metabolites.**

All valerolactones were synthesised as 4-*(RS)-*enantiomeric mixtures. 5-(4’-Hydroxy-3’-methoxyphenyl)-γ-valerolactone (4H3MVL) and some 5-(3’,4’-dihydroxyphenyl)-γ-valerolactone **5** (3,4DHVL) were synthesised as described by Chang *et al.* ^[1]^ In addition, gram quantities of **5** could be made by a modification of this procedure (Figure 1). 2-Triisopropylsilyloxyfuran **1,** ^[2]^ rather than 2-t-butyldimethylsilyloxyfuran ^[1]^ was easier to prepare in larger quantities as it was stable to purification by distillation. And overall yields were more consistent and higher in our hands if, rather than using a one pot synthesis ^[1]^ of 5-[(3,4-dibenzyloxyphenyl)methylene]furan-2-one **4**, the intermediate 2-[(3,4-dibenzyloxyphenyl)-triisopropylsilyloxy-methyl]-2H-furan-5-one **3** was isolated as a diastereomeric mixture and purified before conversion to **5** via **4**.

Figure1

5-(4’-Hydroxyphenyl)-γ-valerolactone 3’-*O*-glucuronide (4HVL3-glucuronide), and 5-(3’-hydroxyphenyl)-γ-valerolactone 4’-*O*-glucuronide (3HGV4-glucuronide) were synthesised as shown in Figures 2 and 3. 3-benzyloxy-4-hydroxybenzaldehyde **7** ^[3]^ was silylated to give 3-benzyloxy-4-(tert-butyldimethylsilyloxy)benzaldehyde **8.** Reaction of **8** with **9** by the general procedure of Chang *et al* ^[1]^ gave 5-[4-(*tert*-butyldimethylsilyl)-3-benzoxybenzylidene]furan-2(5H)-one as a mixture of its Z and E isomers **10a** and **10b**. Hydrogenation of **10a** and **10b** led to debenzylation and reduction as hoped, but also induced *tert*-butyldimethylsilyl group migration between the 3- and 4-hydroxyl groups, to give a mixture of the 3-silylated and 4-silylated products **11** and **12**. This rearrangement was unexpected. Kim *et al ^[4]^* have reported removal of TBS protection groups during treatment with palladium on carbon and hydrogen in methanol; yielding the unprotected alcohol and tert-butyl-methoxy-dimethyl-silane. In the present case, in aprotic acetonitrile attack by the vicinal hydroxyl rather than the solvent would explain TBS-migration. Whatever the mechanism, we recognised this migration as fortuitous, as subsequent synthetic steps should lead to a mixture of both desired glucuronides **18** and **19**. And so it proved. Glucuronidation of the mixture of **11** and **12** with methyl 3,4,5-triacetoxy-1-(2,2,2-trichloroethanimidoyl)-α-D-glucuronate **13** gave the expected products **14a** and **15a**. A pure mixture of the latter isomers was obtained by column chromatography on silica; during chromatography, smaller amounts of the desilylated **14b** and **15b** were formed. De-esterification and desilylation of **14a** and **15a** and **14b** and **15b** were achieved by hydrolysis with methanolic sodium carbonate, and undesired lactone ring-opening was reversed by treatment of **16** and **17** with hydrochloric acid. Finally, pure **18** and **19** were obtained by reverse phase preparative chromatography. (The yields of **18** and **19** from **14a** and **15a** and **14b** and **15b** were lower than anticipated, and these conversions would benefit from further optimisation. In particular, some β-elimination of the protected glucuronic acid groups led to unwanted by-products during base hydrolysis, and some glucuronide hydrolysis led to the formation of **5** was during acid treatment.)

A mixture of 5-(3’-hydroxyphenyl)-γ-valerolactone-3’-*O*-sulfate, ammonium salt **20** (4HGV3-sulfate), 5-(4’-dhydroxyphenyl)-γ-valerolactone-4’-*O*-sulfate, ammonium salt **21** (3HGV4-sulfate) was prepared by direct sulfation of 5-(3’,4’-dihydroxyphenyl)-γ-valerolactone **5** with chlorosulfonic acid. No disulfated product was observed. (In contrast, we found treatment of **5** with 2,2,2-trichloroethyl chlorosulfate (TCECS) ^[5] [6]^ gave exclusively a di-sulfated derivative.) Preparative reverse phase hplc using an ammonium acetate/acetonitrile gradient gave a pure mixture of the two monosulfates after lyophilisation. They eluted as overlapping peaks, but it proved possible to isolate small amounts of the each monosulfate, which were subsequently assigned as 3’- or 4‘-substituted by nmr.

Figure 2

Figure 3

Figure 4

**References**

[1] X. W. Chang, W. M. Peng, Y. F. Yao, J. Koek, *Synth. Commun.* **2010**, *40*, 3346.

[2] S. F. Martin, S. K. Bur, *Tetrahedron* **1999**, *55*, 8905.

[3] M. Zhang, G. E. Jagdmann, M. Van Zandt, P. Beckett, H. Schroeter, *Tetrahedron-Asymmetry* **2013**, *24*, 362.

[4] S. Kim, S. M. Jacobo, C. T. Chang, S. Bellone, W. S. Powell, J. Rokach, *Tetrahedron Letters* **2004**, *45*, 1973.

[5] Y. Liu, I. F. F. Lien, S. Ruttgaizer, P. Dove, S. D. Taylor, *Org. Lett.* **2004**, *6*, 209.

[6] N. Brindani, P. Mena, L. Calani, I. Benzie, S. W. Choi, F. Brighenti, F. Zanardi, C. Curti, D. Del Rio, *Molecular Nutrition & Food Research* **2017**, *61*, 12.

**Experimental.**

**General methods**

Solvents were dried over freshly activated 3 A° molecular sieves. Evaporations were performed in vacuo at 50°C. Solids were dried overnight in vacuo over P_2_O_5_ before use.

NMR spectra were recorded on a Bruker Avance NMR spectrometer (Bruker BioSpin GmbH, Rheinstetten, Germany) equipped with a cryoprobe (TCI) and operating at 600 MHz for ^1^H. Signals were referenced to residual undeuterated solvent. Column chromatography used prepacked silica cartridges (KP-Sil, Biotage) and UV detection. ESI-LCMS used a 5 μm C-18 Phenomenex Lunar column (250 mm × 4.4 mm) eluted at 1 mL/min at 30 °C with a 0.1% formic acid/0.1% formic acid in acetonitrile gradient (for glucuronides), or 50 mM ammonium formate, pH 5/ 50 mM ammonium formate in 90 % acetonitrile (for sulfates), linked to an Agilent 1100 hplc system equipped with an Agilent MSD SL single quadrupole mass spectrometer. Preparative reversed phase chromatography used an Agilent 1200 series system with UV detection, equipped with a 5 μm Phenomenex Luna Axia column (250 mm x 21.2 mm), at a flowrate of 21 ml/min.

**2-[(3,4-dibenzyloxyphenyl)-triisopropylsilyloxy-methyl]-2H-furan-5-one** **3** **as a mixture of diastereomers 3a and 3b.**

Triisopropylsilyl triflate (3.74 g, 3.3 ml, 12.2 mmol) [2] was added dropwise to a stirred solution of 3,4-dibenzyloxybenzaldehyde (7.77 g, 24.4 mmol) and 2-triisopropylsilyloxyfuran **1** ^[2]^ (7.04 g, 29.3 mmol) in dry dichloromethane (120 ml) under argon at -78°. After 4.5 h the temperature was allowed to rise to ambient. The solution was washed with cold brine (2 x 100 ml) and evaporated to 16.57 g of a yellow oil. Purification by column chromatography on silica using a hexane-ethyl acetate gradient gave a mixture of **3a** and **3b** (12.55 g, 92%) as a viscous green-yellow oil. ESI-LCMS: Two close running peaks. No ionisation was observed. **3a** and **3b**  were not further characterised, but were directly converted to **4**.

**5-[(3,4-dibenzyloxyphenyl)methylene]furan-2-one** **4.**

1,8-Diazabicyclo(5.4.0)undec-7-ene (3.55 g,1.05 eq., 3.20 ml) was added dropwise to a solution of **3** (12.4 g, 22.2 mmol) in dry dichloromethane (125 ml). After 2 h the solution was evaporated and purified by column chromatography on silica using a hexane-ethyl acetate gradient to give pure **4**, identical to authentic material. ^[1]^

**5-(3’,4’-dihydroxyphenyl)-γ-valerolactone** **5.**

Hydrogenation of **4** as described gave authentic **5** ^[1]^ in 75% yield after column chromatography on silica using a hexane-ethyl acetate gradient.

**3-benzyloxy-4-hydroxybenzaldehyde** **7** was synthesised as described by Zhang *et al*. ^[3]^

**Synthesis of** **3-benzyloxy-4-(tert-butyldimethylsilyloxy)benzaldehyde** **8.**

A solution of **7** (3.41 g, 14.3 mmol) and triethylamine (2.17 g, 1.5 eq.) in 30 ml dry dichloromethane was stirred at rt. A solution of *tert*-butyldimethylsilyl chloride (3.24 g, 1.5 eq.) in dichloromethane was added dropwise over 5 min. After 5h, water (80 ml) was added, and the mixture was extracted with dichloromethane (2 x 80 ml). The extracts were washed with water (2 x 40 ml) and brine (1 x 40 ml) and dried (MgSO_4_). The crude product was isolated by evaporation and purified by column chromatography on silica using a hexane-ethyl acetate gradient. 3.40 g (67%) of **8** as a pale yellow liquid was obtained. ^1^H NMR (CDCl_3_) δ: 0.13 (s, 6H, 2 x CH_3_Si); 0.96 (s, 9H, (CH_3_)_3_C); 5.10 (s, 2H, PhCH_2_); 6.99 (1H, d, J =8.1 Hz, H-5); 7.34 -7.45 (5H, m, benzyl H); 7.39 (1H, d, J =8.1, 1.7 Hz, H-6); 7.48 (1H, d, J = 1.7 Hz, H-2); 9.84 (1H, s, CHO). ESI-LCMS: *m/z* +343 [M+H^+^].

**Synthesis of Z/E 5-[4-(*tert*-butyldimethylsilyl)-3-benzoxybenzylidene]furan-2(5H)-one 10a + 10b** followed the general procedure of Chang *et al.* ^[1]^ A mixture of, furan-2(5H)-one (0.614 g, 7.13 mmol), powdered 3A molecular sieves and dichloromethane (70 ml) was stirred under Ar at rt for 5 min. *tert*-Butyldimethylsilyl triflate (1.93 g, 1 eq.) was added dropwise over 5 min. After 1h, **7** (2.50 g, 1 eq.) and N,N-diisopropylethylamine (2.3 g, 3 eq.) were added and stirring was continued for 1 h. 1,8-Diazabicyclo[5.4.0]undec-7-ene 2.22 g, 2 eq.) was added. After 1 h 20 min, the mixture was filtered through a silica) 4 x 3 cm column with 1:1 hexane/ethyl acetate (250 ml). After evaporation, the crude material was purified by column chromatography on silica using a hexane-ethyl acetate gradient to yield separately the Z and E product isomers **10a** and **10b** in 0.70 g and 1.09 g yield respectively (60% overall). **10a** ^1^H NMR (CDCl_3_) δ: 0.13 (s, 6H, 2 x CH_3_Si); 0.98 (s, 9H, (CH_3_)_3_C); 5.11 (s, 2H, PhCH_2_); 5.93 (1H, s, H-5); 6.14 (1H, d, J = 5.2 Hz, H-2); 6.87 (1H, d, J = 8.2 Hz, H-5’); 7.20 (1H, dd, J = 8.2, 2.0 Hz, H-6’); 7.32 (1H, t, J = 7.3 Hz, H-4’’); 7.39 (2H, t, J = 7.2 Hz, H-3’’, H-5’’); 7.44 (1H, d, J = 5.2 Hz, H-3); 7.50 (2H, t, J = 7.2 Hz, H-2’’, H-6’’); 7.52 (1H, d, J = 2.0 Hz, H-2’). ESI-MS: *m/z* +409 [M+H^+^]. **10b** 0.16 (s, 6H, 2 x CH_3_Si); 0.98 (s, 9H, (CH_3_)_3_C); 5.10 (s, 2H, PhCH_2_); 6.22 (1H, dd, J = 5.5, 1.8 Hz, H-2); 6.67 (1H, d, J = 1.0 Hz, H-5); 6.83 (1H, d, J = 1.8 Hz, H-2’); 6.86 (1H, dd, J = 8.1, 1.8 Hz, H-6’); 6.88 (1H, d, J = 8.8 Hz, H-5’); 7.34 (1H, t, J = 7.3 Hz, H-4’’); 7.39 (2H, t, J = 7.2 Hz, H-3’’, H-5’’); 7.42 (2H, t, J = 7.2 Hz, H-2’’, H-6’’); 7.45 (1H, d, J = 5.5 Hz, H-3). ESI-LCMS: *m/z* +409 [M+H^+^].

**Preparation of 5-[[3-[****tert-butyl(dimethyl)silyl]oxy-4-hydroxy-phenyl]methyl]- tetrahydrofuran-2-one 11 and 5-[[4-[tert-butyl(dimethyl)silyl]oxy-3-hydroxy-phenyl]methyl]tetrahydrofuran-2-one 12.**

**From 10a.**

**10a** (0.70 g, 2.04 mmol), 10% palladium on carbon (0.2 g) and acetonitrile (40 ml) were stirred under hydrogen (1 atmosphere) at room temperature for 15 h. The mixture was filtered (0.22 um) and the filtrate purified by column chromatography on silica using a hexane-ethyl acetate gradient to give 0.24 g of a mixture of **11** and **12** (36%).

**From 10b.**

**10b** (1.04 g, 2.54 mmol), 10 % palladium on carbon (0.3 g) and acetonitrile (60 ml) were stirred under hydrogen (1 atmosphere) at room temperature for 22 h. The mixture was filtered (0.22 um) and the filtrate purified by column chromatography on silica using a hexane-ethyl acetate gradient to give 0.12 g of a mixture of **11** and **12** (15%).

**11** + **12.** ^1^H NMR (CDCl_3_) δ: 0.26 (s, 12H, 4 x CH_3_Si); 1.00 (9H, (CH_3_)_3_C); 1.01 (9H, (CH_3_)_3_C); 1.88 -1.98 (2H, m, H-3a); 2.20 - 2.28 (2H, m, H-3b); 2.44 - 2.50 (4H, m, 2 x H-a, 2 x H-2b); 2.78 - 2.85 (2H, m, 2 x Ha); 2.92 - 3.00 (2H, m, 2 x Hb); 6.60 (1H, dd, J= 2.2, 8.2 Hz, H-5’ **11**); 6.69 (1H, d, J = 2.1 Hz, H-2’ **12**); 6.71 (1H, dd, J = 2.1, 8.4 Hz, H-5’ **12**); 6.75 (1H, d, J = 8.2 Hz, H-6’ **11**); 6.80 (1H, d, J = 2.2Hz, H-2’ **11**); 6.86 (1H, d, J = 8.4 Hz, H-6’ **12**). ESI-LCMS (both peaks): *m/z* +323 [M+H^+^]; +340 [M+NH_4_^+^]; +345 [M+Na^+^].

**Preparation** **of** **methyl 3',4',5'-triacetyl-6-[2- tert-butyl(dimethyl)silyl]oxy-5-[(5-oxotetrahydrofuran-2-yl)methyl]phenoxy]-beta-D-glucuronate 13a and methyl 3',4',5'-triacetyl-6-[2- tert-butyl(dimethyl)silyl]oxy-4-[(5-oxotetrahydrofuran-2-yl)methyl]phenoxy]-beta-D-glucuronate 14a.**

A mixture of **11** and **12**, **13**, dichloromethane and powdered 3A molecular sieves was stirred, under Ar for 30 min at room temperature. The mixture was cooled to -20°, boron trifluoride etherate (80μl) was added, and stirred for 15 min. The temperature was raised to -10° and stirring was continued for 4 h. Triethylamine (200 μl) was added, and the reaction mixture was filtered, to give a crude mixture of **14a** and **15a**, which were purified by column chromatography on silica using a hexane-ethyl acetate gradient to give 0.63 g (72 %) of a pure mixture of **14a** and **15a**. ESI-LCMS (both peaks): *m/z* +651 [M+NH_4_^+^]; + 661 [M+Na^+^].

**Preparation** **of** **methyl 3',4',5'-triacetyl-6-[2-hydroxy-5-[(5-oxotetrahydrofuran-2-yl)methyl]phenoxy]-beta-D-glucuronate 13b and methyl 3',4',5'-triacetyl-6-[2-hydroxy-4-[(5-oxotetrahydrofuran-2-yl)methyl]phenoxy]-beta-D-glucuronate 14b.**

Silica chromatography of the crude mixture of **14b** and **15b** also produced 0.20 g (26 %) of a pure mixture of the de-silylated products **14b** and **15b**. ESI-LCMS: *m/z* +542 [M+NH_4_^+^]; + 547 [M+Na^+^].

**Preparation of 5-(4’-hydroxyphenyl)-γ-valerolactone 3’-*O*-glucuronide 18, and 5-(3’-hydroxyphenyl)-γ-valerolactone 4’-*O*-glucuronide 19**.

From **14a** and **15a.**

A mixture of **14a** and **15a** (0.63 g, 0.99 mmol) was stirred in methanol (40 ml) at 0°. A solution of sodium carbonate (0.5 M, 22 ml) chilled to 0° was added. Stirring was continued overnight on an ice-water bath, during which the mixture attained room temperature. ESI-LCMS revealed two products, (both peaks) *m/z* +420 [M+NH_4_^+^]; +425 [M+Na^+^], consistent with de-silylated, deesterified, lactone-opened products **16** and **17**. Sufficient Dowex 50W (H^+^ form) was added to, with stirring, lower the pH to ~ 3. The mixture was filtered, the filtrate evaporated, and the resultant solid re-dissolved in 2M HCl (25 ml) and stirred for 4.5 h at room temperature. They were purified as described below.

From **14b** and **15b*.***

A mixture of **14b** and **15b** (0.20 g, 0.38 mmol) was stirred in methanol (15 ml) at 0°. A solution of sodium carbonate (0.5 M, 8.7 ml) chilled to 0° was added. Stirring was continued overnight on an ice-water bath, during which the mixture attained room temperature. ESI-LCMS revealed two main products, (both peaks) *m/z* +420 [M+NH_4_^+^]; +425 [M+Na^+^], consistent with the deesterified, lactone-opened products **16** and **17**. Sufficient Dowex 50W (H^+^ form) was added to, with stirring, re-dissolve precipitated solids and lower the pH to ~ 3. The mixture was filtered, the filtrate evaporated, and the resultant solid re-dissolved in 2M HCl (10 ml) and stirred overnight at room temperature. The crude products were combined with those above and purified by reverse phase preparative chromatography using a 0.1 % formic acid- water-acetonitrile gradient to give **18** (39.6 mg, 10 % overall yield from **11** and **12**) and **19** (34.7 mg, 9 % overall yield from **11** and **12**). ESI-LCMS: (both products) *m/z* +407 [M+Na^+^]; +402 [M+NH_4_^+^]; +385 [M+H^+^].

**Summary of ^13^C and ^1^H nmr data \ δ for compounds 5, 18 and 19.**

|  | **5** | | **18** (3’GlcA) | | **19** (4’GlcA) | |
| --- | --- | --- | --- | --- | --- | --- |
| Atom | H | C | H | C | H | C |
| Lactone |  |  |  |  |  |  |
| 1 | - | 177.06 | - | 177.03 | - | 177.03 |
| 2 | 2.35, 2.44 | 28.16 | 2.34, 2.44 | 28.14 | 2.39, 2.45 | 28.16 |
| 3 | 1.83, 2.14 | 26.63 | 1.85, 2.13 | 26.47, 26.58 | 1.84, 2.15 | 26.67 |
| 4 | 4.61 | 80.73 | 4.65 | 80.44, 80.57 | 4.65 | 80.53 |
| 5 | 2.70, 2.76 | 39.82 | 2.75, 2.84 | 39.82 | 2.76, 2.83 | 39.87 |
| Phenyl |  |  |  |  |  |  |
| 1 | - | 127.47 | - | 127.49, 127.52 | - | 131.88 |
| 2 | 6.61, d 2.2 | 116.68 | 6.93 | 118.0, 118.05 | 6.72 | 117.10, 117.15 |
| 3 | - | 145.0 | - | 144.63, 144.71 | - | 146.75 |
| 4 | - | 143.85 | - | 145.74, 145.77 | - | 143.70, 143.73 |
| 5 | 6.63, d 8.0 | 115.43 | 6.74 | 116.0, 116.02 | 6.94 | 116.72 |
| 6 | 6.47, dd | 119.97 | 6.75 | 123.95, 124.09 | 6.61 | 120.05,120.09 |
| GlcA |  |  |  |  |  |  |
| 1 |  |  | 4.80, 4.83 | 101.92, 101.77 | 4.78 | 101.77, 101.92 |
| 2 |  |  | 3.30 | 73.07 | 3.30 | 73.07 |
| 3 |  |  | 3.29 | 75.34 or 75.37 | 3.29 | 75.24 or 75.32 |
| 4 |  |  | 3.38 | 71.46 | 3.37 | 71.46 |
| 5 |  |  | 3.80 | 75.34 or 75.37 | 3.79 | 75.24 or 75.32 |
| 6 |  |  | - | 170.29 | - | 170.26 |

Solvent DMSOd_6_ (^1^H = 2.49, ^13^C = 39.5)

Some atoms have two ^13^C chemical shifts because of the chiral centre at lactone atom 4 coupled with the additional chiral centres from the GlcA.

Relevant Hmbc and Hsqc links:

**5**

Phenyl-lactone: 6.61, 6.47 linked to 39.82, (6.63 not linked): 6.61 and 6.47 are *ortho* to the lactone group

4.61 (H4 lactone) linked to 127.47 (gives C1 of Ph group); also linked to 177.06 (C1 of lactone) – gives quaternary chem shifts.

Within Ph group: 6.63 (H5) linked to 127.47 (C1); 6.61 (H2) to 119.97 (C6); 6.47 (H6) to 116.68 (C2) (all ^3^J couplings).

6.63 and 6.61 both linked to both 143.85, 145.0 (^2^J or ^3^J couplings). 6.47 linked only to 143.85, not 145.0. Conclusion C4 is 143.85; C3 is 145.0 (would be ^4^J from H6, 6.47). Should be possible to use these links to identify C3 and C4 in GlcA compounds.

**18**

The multiplet shape indicated that H2 was downfield at 6.93 ppm. However the near coincidence of chemical shifts for H5 and H6 and their consequent strong coupling meant that they could not be assigned simply on the basis of multiplet shape as in the other ^1^H spectra. Joint examination of the hmbc and hsqc spectra indicated that H6 was at 6.75 and H5 at 6.74 ppm. In particular H5 had an hmbc link to the Ph C1 at 127.5 ppm and H2 and H6 had the hmbc links to C6 and C2 respectively, as described above.

H2 was linked to both C3 and C4 (144.7, 145.8) but 6.75 (H6) was linked only to 145.8 and 6.74 (H5) to 144.7. Assuming these links are due to ^3^J coupling we identify C3 as 144.7 and C4 as 145.8.

H1 (GlcA) has 2d at ~4.80 ppm because of the chiral centre effect. Both these d have hmbc links to the signals at 144.7 (not 145.8). *Conclusion: GlcA is linked at C3.*

Note also the consistent downfield displacement of ^1^H shifts for protons *ortho* to the GlcA substitution site. In both cases the shifts are displaced by ~0.3 ppm from ~6.60 (34DHVL) to ~6.90, affecting H5 in **19** (4GlcA) and H2 in **18** (3GlcA).

**19**

6.72 (Ph H2) and 6.61 (H6) linked to 39.87 (VL C5), 6.94 (H5) not linked.

6.72 (H2) and 6.94 (H5) linked to both 143.7 and 146.75 (C4 and C3 –OH quaternaries); 6.61 (H6) linked only to 143.7: C4 must be 143.7.

4.78 (H1 GlcA) is linked to 143.7, not 146.75. *Conclusion: GlcA is linked at C4.*

**Synthesis of 5-(3’-hydroxyphenyl)-γ-valerolactone-4’-*O*-sulfate, ammonium salt 20, 5-(4’-dhydroxyphenyl)-γ-valerolactone-3’-*O*-sulfate, ammonium salt 21.**

A solution of chlorosulfonic acid (246 mg, 140 ul, 1.1 eq) in dry MeCN (3 ml) was added dropwise to a stirred mixture of **5** (0.40 g, 1.92 mmol) and pyridine (545 ul, 3.5 eq.) under argon in dry acetonitrile (20 ml), cooled to -78°. The mixture warmed gradually to room temperature overnight. The mixture was evaporated, and the residue dissolved in 25 ml 20 % aqueous methanol, filtered, and purified by reverse phase preparative chromatography using a 50 mM ammonium formate, pH 5/ 50 mM ammonium formate in 90 % acetonitrile gradient to give, after lyophilisation to constant weight, pure **20** (15.73 mg, 3%), **21** (1.07 mg 0.2 %), and a pure mixture of **20** and **21** (65.44 mg,11%).

ESI-LCMS: (both products) *m/z* -287 [M-H^-^].

**Summary of ^13^C and ^1^H nmr data \ δ for compounds 5, 20 and 21.**

|  | **5** | | **20** (4’S) | | **21** (3’S) | |
| --- | --- | --- | --- | --- | --- | --- |
| Atom | H | C | H | C | H | C |
| Lactone |  |  |  |  |  |  |
| 1 | - | 177.06 | - | nd | - | nd |
| 2 | 2.35, 2.44 | 28.16 | 2.40, 2.60 | 31.24 | 2.40, 2.60 | 31.22 |
| 3 | 1.83, 2.14 | 26.63 | 2.05, 2.37 | 28.88 | 2.05, 2.37 | 28.72 |
| 4 | 4.61 | 80.73 | 4.95 | 85.51 | 4.95 | 85.62 |
| 5 | 2.70, 2.76 | 39.82 | 2.99, 3.02 | 42.31 | 2.99, 3.02 | 41.80 |
| Phenyl |  |  |  |  |  |  |
| 1 | - | 127.47 | - | nd | - | nd |
| 2 | 6.61, d 2.2 | 116.68 | 6.95, d 1.8 | 120.87 | 7.30, d 1.9 | 126.54 |
| 3 | - | 145.0 | - | nd | - | nd |
| 4 | - | 143.85 | - | nd | - | nd |
| 5 | 6.63, d 8.0 | 115.43 | 7.31, d 8.4 | 125.52 | 7.00, d 8.0 | 119.86 |
| 6 | 6.47, dd | 119.97 | 6.88, dd | 124.36 | 7.10, dd | 130.93 |

Solvent D_2_O (^1^H = 4.79)

nd not determined

**Legends**

Figure 1. Revised synthesis of **5** (3,4DHVL).

Figure 2. Synthesis of (4HVL3-glucuronide) **18**, and (3HGV4-glucuronide) **19**. Part 1.

Figure 3. Synthesis of (4HVL3-glucuronide) **18**, and (3HGV4-glucuronide) **19**. Part 2.

Figure 4. Synthesis of **20** (3HGV4-sulfate) and **21** (4HGV3-sulfate).
